# T cell dysfunction in elderly ARDS patients based on miRNA and mRNA integration analysis

**DOI:** 10.3389/fimmu.2024.1368446

**Published:** 2024-03-20

**Authors:** Yumi Mitsuyama, Hisatake Matsumoto, Yuki Togami, Sayaka Oda, Shinya Onishi, Jumpei Yoshimura, Arisa Murtatsu, Hiroshi Ito, Hiroshi Ogura, Daisuke Okuzaki, Jun Oda

**Affiliations:** ^1^ Department of Traumatology and Acute Critical Medicine, Osaka University Graduate School of Medicine, Osaka, Japan; ^2^ Department of Acute Medicine and Critical Care Medical Center, Osaka National Hospital, National Hospital Organization, Osaka, Japan; ^3^ Department of Oral and Maxillofacial Surgery, Osaka University Graduate School of Dentistry, Osaka, Japan; ^4^ Genome Information Research Center, Research Institute for Microbial Diseases, Osaka University, Osaka, Japan

**Keywords:** ARDS, miRNA, mRNA, PD-1, PD-L1, Th1, Th2

## Abstract

**Background:**

Acute respiratory distress syndrome (ARDS) is respiratory failure that commonly occurs in critically ill patients, and the molecular mechanisms underlying its pathogenesis and severity are poorly understood. We evaluated mRNA and miRNA in patients with ARDS and elucidated the pathogenesis of ARDS after performing mRNA and miRNA integration analysis.

**Methods:**

In this single-center, prospective, observational clinical study of patients with ARDS, peripheral blood of each patient was collected within 24 hours of admission. Sequencing of mRNA and miRNA was performed using whole blood from the ARDS patients and healthy donors.

**Results:**

Thirty-four ARDS patients were compared with 15 healthy donors. Compared with the healthy donors, 1233 mRNAs and 6 miRNAs were upregulated and 1580 mRNAs and 13 miRNAs were downregulated in the ARDS patients. For both mRNA and miRNA-targeted mRNA, canonical pathway analysis showed that programmed death-1 (PD-1) and programmed cell death ligand 1 (PD-L1) cancer immunotherapy pathway was most activated and the Th2 pathway was most suppressed. For mRNA, the Th1 pathway was most suppressed. miR-149-3p and several miRNAs were identified as upstream regulators.

**Conclusion:**

miRNAs regulated the PD-1 and PD-L1 cancer immunotherapy pathway and Th2 pathway through miRNA interference action of mRNA. Integrated analysis of mRNAs and miRNAs showed that T cells were dysfunctional in ARDS patients.

## Introduction

Acute respiratory distress syndrome (ARDS) is a common cause of respiratory failure in critically ill patients and is defined by the acute onset of noncardiogenic pulmonary edema, hypoxemia, and the need for mechanical ventilation (1). ARDS is often caused by pneumonia, sepsis, aspiration of stomach contents, or severe trauma and occurs in approximately 10% of intensive care unit (ICU) patients worldwide. Mortality rate remain high, ranging from 30–40% in most studies (2). Despite recent medical advances, treatment of ARDS remains a challenge. There is no effective drug therapy, and supportive care such as low tidal volume ventilation, supine positioning, conservative fluid management, and neuromuscular blockade for ventilator dyssynchrony are the mainstays of therapy. Underlying the difficulty of fundamental treatment is the lack of a well-defined molecular mechanism of ARDS.

Progress in transcriptome research, which comprehensively understands the expression status of genes in living organisms, is revealing the functions and regulatory mechanisms of genes that are unknown in various diseases. microRNAs (miRNAs), which consist of around 22 bases, are a type of non-coding RNA. miRNAs regulate gene expression by binding to the 3′ untranslated region of mRNA (coding RNA) in a complementary manner (3). It is estimated that 30–50% of all genes are regulated by miRNAs. Several miRNAs have been reported to be associated with outcome in ARDS (4, 5). Systematic analysis of miRNA and mRNA expression profiling will provide insight into understanding the molecular mechanisms of ARDS. Thus, the aim of this study was to elucidate the role of miRNAs in the pathophysiology of ARDS by combining miRNA and mRNA sequencing analysis.

## Materials and methods

### Study design and participants

This single-center, prospective, observational clinical study was conducted in patients with ARDS admitted to the Department of Traumatology and Acute Critical Medicine, Graduate School of Medicine, Osaka University between July 2020 and February 2021. Eligible patients were those 18 years of age or older who were directly admitted to the hospital for ARDS or who were evaluated by a clinician for admission to the ICU and transferred from another hospital. Patients who were lactating, pregnant, had malignancies undergoing treatment, or who refused to participate in the study were excluded. The diagnosis of ARDS followed the Berlin definition (1). The diagnosis of acute exacerbation of interstitial pneumonia followed that in a previous report (6). Clinical and biological parameters such as demographic characteristics, duration of mechanical ventilation and hospitalization, and comorbidities were collected from the electronic medical record. Severity scores were recorded using the Acute Physiology and Chronic Health Evaluation (APACHE) II score (range 0–71) and Sequential Organ Failure Assessment (SOFA) score (range 0–24), with ARDS severity rated as mild, moderate, or severe (7, 8). This study involving humans was approved by the institutional review board of Osaka University Hospital (approval number: 885 [Osaka University Critical Care Consortium Novel Omix Project; Occonomix Project]). Healthy donors had no disease under treatment and were recruited from the public. Written informed consent was obtained from all patients and the healthy donors. The study was conducted in accordance with the Declaration of Helsinki.

### Sample preparation and isolation of RNA

The peripheral blood of each patient was collected within 24 hours of admission using a PAXgene™ Blood RNA Tube (BD Biosciences, San Jose, CA), and total RNA was isolated using a PAXgene Blood RNA Kit (BD Biosciences) according to the manufacturer’s instructions. Quantitative measurement of total RNA was performed using a Nano Drop One (Thermo Fisher Scientific, Waltham, MA), and qualitative measurement was performed using an Agilent 2100 bioanalyzer (Agilent, Santa Clara, CA). The quality of the isolated RNA is shown in **Supplementary Table S1**. All blood samples for analysis were collected in collection tubes and stored at -30°C until further analysis. For the healthy donors, blood was collected in the same manner and RNA was isolated.

### Library preparation, RNA sequencing, and RNA-seq analysis

The amount of RNA input for library preparation was 200 ng of total RNA for mRNA-Seq and 100 ng of total RNA for miRNA-Seq. After total RNA isolation, full-length cDNA was prepared using a SMART-Seq HT kit (Takara Bio, Mountain View, CA) according to the manufacturer’s instructions. Illumina libraries were prepared using a Nextera DNA Library Preparation Kit (Illumina) according to the SMARTer kit instructions. DNA libraries were converted to libraries for DNBSEQ using the MGIEasy Universal Library Conversion Kit (App-A). Sequencing was performed on the DNBSEQ-G400RS platform in 2 × 100-bp paired-end mode. RNA-Seq analysis was performed with some modifications and changes from the analysis reported in a previous article (9). The sequenced reads were mapped to the human reference genome sequences (hg19) using TopHat (ver. 2.2.1), in combination with Bowtie2 (ver. 2.2.8), and SAMtools (ver. 0.1.18). Raw read counts of gene-level expression for each gene-sample combination were calculated using featureCounts in the subread-2.0.0 package. The mRNAs targeted by miRNA (miRNA-targeted mRNAs) were identified by in silico analysis using miRNA Target Filter^®^ (QIAGEN) for predicted miRNA-targeted mRNA interactions.

Micro RNA-Seq analysis was performed with some modifications and changes from the analysis reported in a previous article (9). Small RNA libraries were constructed using the NEBNext Small RNA Library Prep Set for Illumina (New England Biolabs, Ipswich, MA) according to the manufacturer’s instructions and sequenced with 101-bp single-end reads on a NovaSeq 6000 platform (Illumina). Prior to analysis of the small RNA-Seq data, reads were trimmed of the 3′ adapter sequence (AGATCGGAAGAGCACACGTCT) with Cutadapt ver. 3.2. Mapping and quantification were performed with miRDeep2 software ver. 2.0.1.3 according to the following two steps. In the first step, the script named collapse_reads_md.pl of miRDeep2 summarized same read sequences in each fasta file to one sequence with number of reads as an associated annotation. In the second step, the script named quantifier.pl mapped the summarized sequences to the miRBase ver. 22.1 human miRNA dataset and the RIKEN FANTOM5 atlas of the human miRNAs dataset and quantified expression counts (10). Normalization of the raw count data was performed with DESeq2 ver. 1.34.0. After normalization, normalized counts less than 1 were replaced with 1 to avoid errors caused by dividing by zero or computing the logarithm of zero. Fold change was calculated based on the replaced normalized count. We calculated the Student *t*-test p-value using the logarithm with a base of 2 of the replaced normalized counts.

### Statistical analysis

The number of samples required to extract differentially expressed genes between the patient group and the healthy donors was calculated to be at least 10 cases for 90% power with measured RNA of 27,000, | log2 fold change | of 1.2, and a false discovery rate (FDR) of 0.1 (**Supplementary Figure S1**). Raw count data for mRNA and miRNA were normalized using Integrated Differential Expression and Pathway analysis ver. 0.96 (iDEP.96; http://bioinformatics.sdstate.edu/idep, accessed on 1 August 2023) (11). A cutoff value of at least 0.5 counts per million in five libraries was used for gene selection. A limma-voom analysis was performed to search for differences in gene expression between the ARDS patients and healthy donors (12). Principal component analysis was performed using normalized mRNA and miRNA values to compare gene expression between the ARDS patients and healthy donors. Volcano plot analysis was used to visualize significant changes in the expression list (VolcaNoseR; https://goedhart.shinyapps.io/VolcaNoseR/, accessed on 1 August 2023) (13). Significance was defined as |log2 fold change| > 1.2 and FDR < 0.1. The raw count data for miRNAs were processed in the same manner. To evaluate functional characteristics of RNA expression and upstream regulators of the mRNAs, we analyzed the data by Ingenuity Pathway Analysis (IPA Fall release 2022) (QIAGEN, https://digitalinsights.qiagen.com/products-overview/discovery-insights-portfolio/analysis-and-visualization/qiagen-ipa/). We used miRNA targets predicted from predicted and experimentally observed targets to detect miRNA-mRNA interactions. Adjusted p-values were calculated by Z scores and Fisher’s exact test with multiple comparison test correction. The Z score predicts the activation status of an upstream regulator by the RNA expression pattern of the downstream state of that regulator, and normal pathways and upstream regulators were considered activated if the |Z score| was > 2 and p < 0.05. We analyzed the data by upstream regulator analysis. Canonical pathway analysis (CPA) was performed using significantly different mRNA and miRNA expression to describe specific relationships between RNAs. To investigate enrichment analysis of differentially expressed genes in the ARDS patients, Kyoto Encyclopedia of Genes and Genomes (KEGG) pathway and Gene Ontology (GO) analyses were performed using the web tool ShinyGO (ShinyGO 0.77; http://bioinformatics.sdstate.edu/go/, accessed on 1 August 2023).

Continuous variables are shown as the median and interquartile range (IQR), and categorical variables are shown as frequencies and percentages. The Wilcoxon rank-sum test was used to test continuous variables, and Fisher’s exact test was used to test the nominal variables. All statistical analyses were performed using JMP Pro17 (SAS Institute Inc., Cary, NC, USA).

## Results

### Patient characteristics

Thirty-four ARDS patients and 15 healthy donors were included in the study. Patient characteristics are shown in **Table 1**. The median age of the ARDS patients was 73 years (IQR: 66–79 years). Among them, 44.1% had hypertension, 38.2% had diabetes, and 17.6% had a history of chronic obstructive pulmonary disease. The causative disease of ARDS was Coronavirus disease 2019 (COVID-19) in 73.5%, acute exacerbation of interstitial pneumonia in 14.7%, and bacterial pneumonia in 8.8%. The median APACHE II score of the patients was 14 (IQR: 10–17) and the median SOFA score was 6 (IQR: 3–6). Severe ARDS was present in 14.7% of the patients, and 47.1% had moderate ARDS. The median length of mechanical ventilation was 14 days (IQR: 7–23 days), and 8.8% of the ARDS patients died in hospital.

**Table 1:** Characteristics of the population.

	ARDS	Healthy donors	p value
	n=34	n=15	
Demographics			
Age (years), median (IQR)	73 (66-79)	55 (37-60)	<0.01
Sex, male (%)	23 (67.6)	8 (53.3)	0.36
BMI, median (IQR)	22.6 (20.7-25.4)	21.7 (20.7-23.2)	0.39
Comorbidities, (%)			
Hypertension	15 (44.1)	1 (6.7)	0.02
Diabetes	13 (38.2)	1 (6.7)	0.04
Chronic obstructive pulmonary disease	6 (17.6)	0 (0)	0.16
Renal insufficiency	6 (17.6)	0 (0)	0.16
Immunocompromise	5 (14.7)	0 (0)	0.31
Malignant neoplasm^*^	1 (2.9)	0 (0)	1
Cardiovascular compromise	3 (8.8)	0 (0)	0.54
Diseases causing ARDS			
COVID-19	25 (73.5)		
Clade			
20B	17 (68.0)		
unknown	8 (32.0)		
Exacerbation of interstitial pneumonia	5		
Bacterial pneumonia	3		
Alveolar hemorrhage	1		
Severity of disease on admission			
APACHEII score, median (IQR)	14 (10-17)		
SOFA score, median (IQR)	6 (3-6)		
Severity of ARDS, n (%)			
Severe ARDS, n (%)	5 (14.7)		
Moderate ARDS, n (%)	16 (47.1)		
Mild ARDS, n (%)	13 (38.2)		
Treatment^**^			
Steroid	21 (61.8)		
Antibiotic	8 (23.5)		
Remdesivir	5 (14.7)		
Tocilizumab	2 (5.9)		
Disease course			
Length of mechanical ventilation, days, median (IQR)	14 (7-23)		
Length of stay in hospital, days, median (IQR)	22 (13-43)		
Hospital mortality	3 (8.8)		

ARDS, Acute Respiratory Distress Syndrome; IQR, interquartile range; BMI, Body Mass Index.

COVID-19, coronavirus disease-19.

APACHE, Acute Physiology and Chronic Health Evaluation; SOFA, Sequential Organ Failure Assessment.

*Include post-treatment and follow-up conditions without recurrence.

**Treatments performed prior to the time of sample collection, including treatments performed by previous hospitals.

### Gene expression comparison

**Figure 1:**
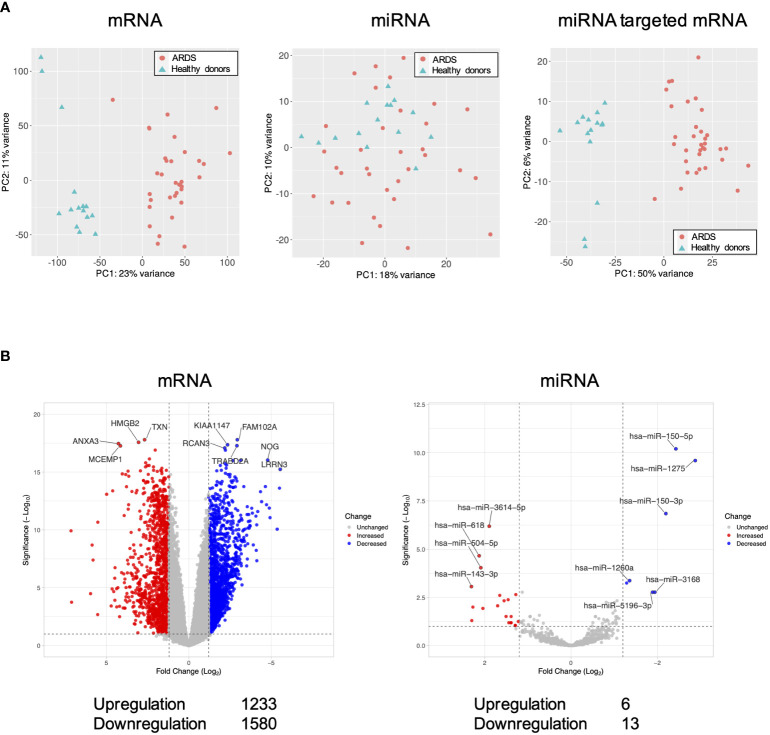
Volcano plots and principal component analysis of mRNA-, miRNA-, and miRNA-targeted mRNA expression in ARDS patients. **(A)** Principal component analysis of all mRNA-, miRNA-, and miRNA-targeted mRNA expression in ARDS patients compared to healthy donors. **(B)** Volcano plot of differential mRNA and miRNA expression in ARDS patients compared to healthy donors. Of the differentially expressed RNAs, 1123 mRNAs and 6 miRNAs were upregulated and 1580 mRNAs and 13 miRNAs were downregulated. The vertical dotted line represents |log2 fold change| >1.2. The horizontal dotted line indicates the threshold for FDR < 0.1. Red dots indicate upregulated RNAs and blue dots indicate downregulated RNAs.

Differentially expressed genes in the ARDS patients are shown in **Figure 1**. Principal component analysis showed that mRNAs and miRNA-targeted mRNAs of the ARDS patients were distinguishable from those of the healthy donors, but miRNAs were not (**Figure 1A**). Of the genes from the ARDS patients, 14,133 mRNAs, 1063 miRNAs, and 1347 miRNA-targeted mRNAs were available for analysis. Compared to the healthy donors, the ARDS patients had increased expression of 1233 mRNAs and 6 miRNAs and decreased expression of 1580 mRNAs and 13 miRNAs (FDR < 0.1, |log2 fold change| > 1.2) (**Figure 1B**).

### Canonical pathway analysis and upstream regulators analysis

**Figure 2:**
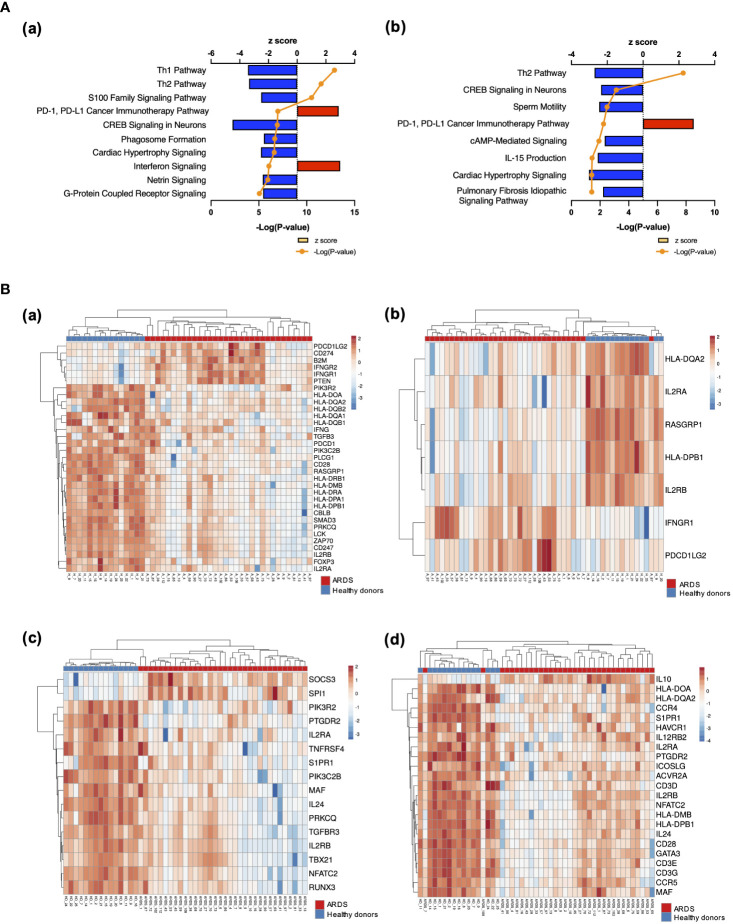
Canonical signaling pathways and heat maps of gene expression. **(A)** Top canonical signaling pathways with p value < 0.05 of mRNA in ARDS patients identified using IPA (a) Top 10 activated and inhibited canonical signaling pathways of mRNA (b) Top activated and inhibited canonical signaling pathways of miRNA targeted mRNA. **(B)** Heat map of gene expression. (a) Heat map of gene expression involved in PD-1 and PD-L1 cancer immunotherapy signaling. Pathways calculated by RNA-Seq in mRNA. (b) Heat map of gene expression involved in the PD-1 and PD-L1 cancer immunotherapy signaling pathway calculated by RNA-Seq in miRNA-targeted mRNAs. (c) Heat map of gene expression involved in the Th2 pathway. (d) Heat map of gene expression involved in the Th2 pathway calculated by RNA-Seq in miRNA-targeted mRNAs.

**Figure 3:**
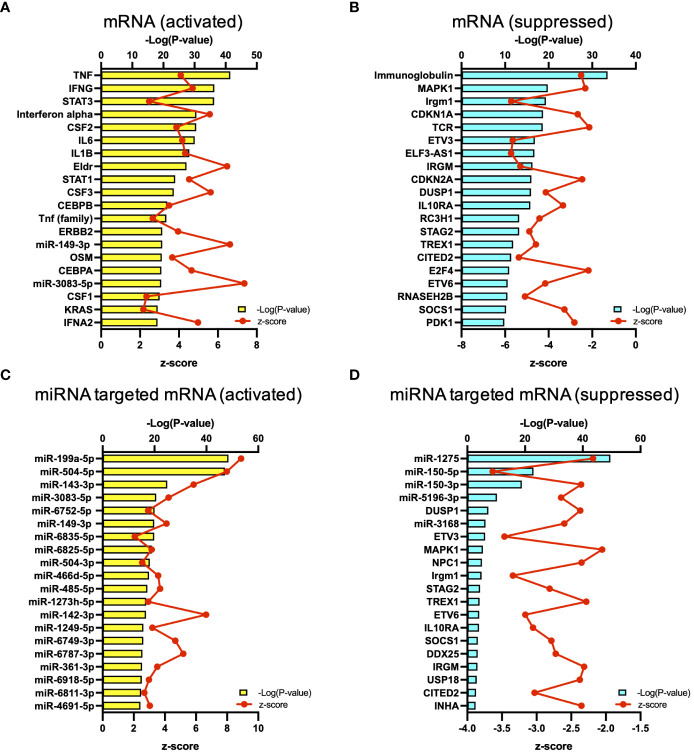
Upstream analysis. **(A)** Top 20 activated upstream regulators in mRNAs. **(B)** Top 20 inactivated upstream regulators in mRNA. **(C)** Top 20 activated upstream regulators in the miRNA-targeted mRNAs. **(D)** Top 20 inactivated upstream regulators in the miRNA-targeted mRNAs.

**Figure 4:**
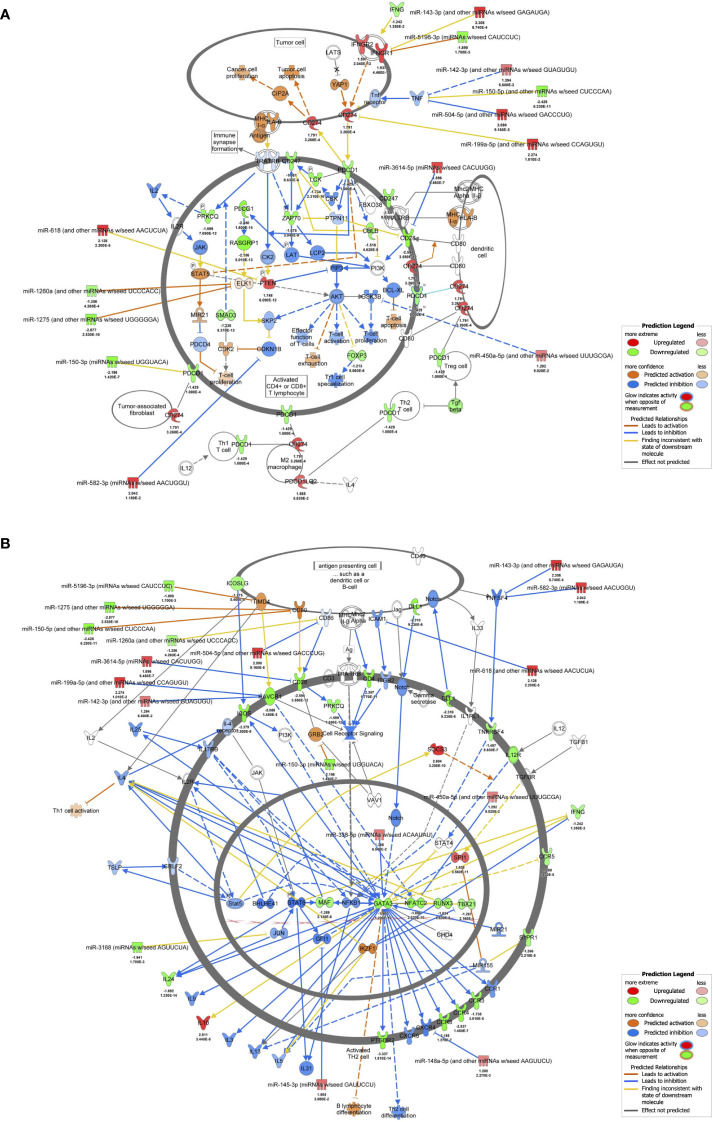
mRNA-miRNA integration analyses. **(A)** mRNA-miRNA integration analysis of PD-1 and PD-L1 cancer immunotherapy pathways predicted by IPA. mRNAs and miRNAs with FDR < 0.1, |log2 fold change| > 1.2 were included in the activated PD-1 and PD-L1 cancer immunotherapy pathway. **(B)** mRNA-miRNA integration analysis of Th2 pathway predicted by IPA. mRNAs and miRNAs with FDR < 0.1, |log2 fold change| > 1.2 were included in the activated Th2 pathway.

The results of RNA-seq were submitted to CPA by IPA to list the canonical signaling pathways activated in ARDS. CPA predicted that 18 mRNA pathways were activated while 29 mRNA pathways were inhibited (|Z score| > 2; p value of overlap < 0.05) (**Supplementary Table S2**). CPA further predicted that one miRNA-targeted mRNA pathway was activated while 7 miRNA-targeted mRNA pathways were inhibited (|Z score| > 2; p value of overlap < 0.05) (**Supplementary Table S3**). The top pathways with a p value < 0.05 are shown in **Figure 2A**. According to changes in the levels of mRNAs and miRNA-targeted mRNAs, the programmed death-1 (PD-1) and programmed cell death ligand 1 (PD-L1) cancer immunotherapy pathway was the most activated in these analyses, whereas the Th2 pathway was the most inhibited pathway. The cAMP-responsive element-binding protein (CREB) signaling in neurons and cardiac hypertrophy signaling were inhibited. To validate the results of CPA by IPA, KEGG pathway and GO analyses were performed on mRNAs and miRNA-targeted mRNAs of the ARDS patients. The analyses predicted activation of the PD-1 and PD-L1-related pathway (**Supplementary Figure S2**). The PD-1 and PD-L1 cancer immunotherapy pathway contained 33 mRNAs and 11 miRNA-targeted mRNAs (**Figure 2B**). Both mRNA and miRNA-targeted mRNA contained PDCD1LG2 and IFNGR1, and gene expression of both was elevated in the ARDS patients. The Th2 pathway contained 47 mRNAs and 23 miRNA-targeted mRNAs (**Figure 2B**). Upstream regulators analysis identified 1251 activated and 118 inhibited potential upstream regulators in the ARDS patients (p value of overlap < 0.05). These top 20 upstream regulators are shown in **Figure 3**. CPA by IPA using mRNAs showed the predicted relationship between RNAs in the activated PD-1 and PD-L1 cancer immunotherapy pathway and the inhibited Th2 pathway (**Figure 4**). In the PD1 and PD-L1 cancer immunotherapy pathway, downregulation of CD247, PDCD1, and CD28 gene expression and upregulation of CD274 and miR-3614-5p were measured. The upregulated miR-618 was associated with increased expression of the Phosphatase and tensin homolog (PTEN) gene. In the Th2 pathway, miR-338-5p predicted suppression of GATA3 gene expression and miR-450a-5p predicted suppression of Nuclear factor of activated T cells 2 (NFATC2) gene expression. This then predicted a downregulation of interferon (IFN) gamma and IL-4 gene expression and an upregulation of IL-10 gene expression. We observed downregulation of the gene expression of CD28 and CD4, which comprise T cell receptor (TCR) signaling. miR-3614-5p, which inhibits the gene CD28, was also measured.

## Discussion

ARDS is a form of noncardiogenic pulmonary edema associated with a systemic inflammatory condition and is characterized by diffuse alveolar damage (2). The pathogenesis of ARDS is multifactorial and involves diverse immune cells of the innate and adaptive immune systems, and these injuries cause lung injury (14). This study revealed that regulation by miRNAs in patients with acute ARDS resulted in activation of the PD-1 and PD-L1 pathway and suppression of the Th2 pathway. To our knowledge, there have been no integrated mRNA-miRNA analysis studies in ARDS. Our findings indicate that patients with ARDS have decreased antigen-presenting capacity and T cell refractoriness to antigen presentation, as well as T cell dysfunction, from early onset of the disease.

The presentation of antigen information from antigen-presenting cells to T cells occurs through the interaction of Human Leukocyte Antigen (HLA) on antigen-presenting cells and TCR on helper T cells. In particular, HLA class II (HLA-DR, DQ, DP, etc.) presents foreign antigens taken up by phagocytosis to helper T cells. Mirchandani et al. reported that HLA-DR gene expression was downregulated in monocytes from ARDS patients, as were four MHC complex genes (HLA-DMA, HLA-DMB, HLA-DQA1, and HLA-DRB3) important for antigen presentation function (15). The present study also showed downregulation of these HLA antigens, and HLA-DQ2 and HLA-DPB1 were also downregulated (**Figure 2B**).

The activation of T cells requires the transduction of a co-signal in which B7 molecules on antigen-presenting cells (e.g., CD80 and CD86) bind to CD28 family molecules on T cells as ligands, in addition to the main signal of binding between HLA and the TCR. The CD28 family molecules include those that stimulate and those that inhibit T cell activation, such as CD28 proteins and ICOS (inducible costimulatory molecules) proteins, the latter of which include PD-1 and CTLA-4 (cytotoxic T lymphocyte antigen-4) (16).

First, regarding the main signal, upstream regulator analysis in this study showed downregulation of TCR expression and downregulation of gene expression of CD4, an auxiliary receptor of TCR, and CD247, a component of TCR, was also measured (**Figures 3**, **4**). Dysfunction of T cells by downregulation of gene CD247 has been shown in a variety of chronic conditions, including cancer, autoimmune diseases, and infectious diseases (17). Its immuno-epidemiological background is described as persistent exposure to antigens and development of chronic inflammatory responses, and it has been reported that downregulation of gene CD247 expression correlates with mortality in septic patients (18, 19). Liao and Liao reported that CD247 is a hub gene for ARDS, which is consistent with our results (20).

Next, regarding the co-stimulatory signals, in this study, miR-3614-5p suppressed the gene expression of CD28, and downregulation of the gene expression of ICOS was measured (**Figure 4B**). miR-3614-5p, which has been reported as an antiviral miRNA in dengue virus infection (21), is induced in immune cells by type I IFN and targets adenosine deaminases acting on RNA-1 (ADAR1) to indirectly edit the viral genome (22). In the present study, 73.5% of the patients had viral infections. Given that IFNα was an important upstream regulator, MiR-3614-5p may have been induced by IFN and exerted its antiviral effect via downregulation of CD28 (**Figure 3**).

Finally, regarding co-inhibitory signals, activation of the PD-1 and PD-L1 cancer immunotherapy pathway was shown in this study (**Figure 2A**). The PD-1/PD-L1 axis, which suppresses T cell activation, regulates the balance between biological defense and immune tolerance by inhibiting excessive lymphocyte activation (23). Lymphocytes with high PD-1 expression are suppressed in differentiation and proliferation, resulting in differentiation into Th2 cells that secrete IL-10 and other anti-inflammatory cytokines, ultimately leading to a dysfunctional state known as exhausted lymphocytes. Gene expression of both PD-1 and PD-L1 was reported to be upregulated in ARDS due to sepsis, and CD8 dysfunction was also observed (24). In the present study, the PD-1 and PD-L1 cancer immunotherapy pathway was activated in both mRNAs and miRNA-targeted mRNAs (**Figure 2A**). In addition, we observed upregulation of PTEN gene expression, which negatively regulates the phosphoinositide 3-kinase (PI3-kinase) pathway (**Figure 3A**). PTEN is known to play a role in innate immunity by activating Interferon Regulatory Factor 3 (IRF3), the master transcription factor for type I IFNs, and maintaining T cell responses via PI3-kinase (25, 26). Many of the patients in this study had severe COVID-19, and IFN signaling has been reported to be severely impaired in critically ill patients (27). Samples in this study were collected early in the infection, within 24 hours of admission, which may differ from the stage of disease in previous studies. In addition, many patients in this study had undergone therapeutic interventions prior to sample collection, which may have affected the IFN pathway. It has been reported that PTEN is upregulated in ARDS patients due to COVID-19 and that it promotes inflammation in a model of acute lung injury (28–30). Finally, the suppression of TCR by inhibiting the co-stimulatory signals and enhancing the co-inhibitory signals resulted in a state of refractoriness of helper T cells to stimuli from antigen-presenting cells.

T cell dysfunction indicates suppression of B cell activation by helper T cells, and decreased expression of HLA antigen genes leads to decreased antigen presentation by B cells to T cells (**Figure 2B**) (31). As immunoglobulins were the most suppressed in the analysis of the upstream regulators, T cell dysfunction can lead to suppression of acquired immunity (**Figure 3B**). Persistence of T cell dysfunction is associated with secondary infections and hospital mortality, but importantly, T cell dysfunction is reversible (32, 33). In a clinical trial of anti-PD-L1 antibody in septic patients, an improvement in immune status was observed with increased gene expression of HLA-DR in monocytes beyond 28 days (34). Given the similarities in immunological backgrounds of both ARDS and sepsis patients, there may be a potential benefit of anti-PD-L1 antibody therapy for ARDS patients as there is considerable overlap in mRNAs and miRNAs that are differentially expressed in diseased and healthy individuals (35).

There have been various reports on the role of miRNAs in ARDS, and miR-150-3p has been reported to have a protective role against the pathogenesis of ARDS by reducing cell apoptosis, autophagy, and the release of inflammatory cytokines (36, 37). However, there are no previous reports on the effects of miR-150-3p on the PD-1 gene or of miR3614-5p on the CD28 gene that were observed in the present study. miR-149-3p, which was shown to be the upstream regulator in the present study, has been reported to be associated with T cell dysfunction in breast cancer cells (38). In CD8+ T cells overexpressing PD-1, miR-149-3p bound to mRNA encoding PD-1, and administration of miR-149-3p resulted in T cell proliferation and secretion of cytokines such as IL-2, TNF-α, and IFN-γ. In cancer and genetic diseases, genomic drug discovery using genes as therapeutic targets is in progress (39). The mRNAs and miRNAs identified in this study might lead to new drug discovery by elucidating the mechanisms in future basic research.

## Limitations

This study has several limitations. First, the age difference between the patients and the healthy donors is the most important limitation of this study. The efficacy of the immune response declines with age (40). In the elderly, expression of the CD28 gene, which was differentially expressed in the patients of this study compared to that in the healthy donors, is reduced, whereas increased expression of the PD-1 and CD274 genes has been reported (41, 42). It is possible that these changes in gene expression occurred prior to disease onset. Second, the patient background was not uniform, and treatment and medical history may have influenced the results of the analysis. As 61.8% of the patients received steroids, their anti-inflammatory effect on immune cells could have influenced the results. Among the study population, 44.1% had hypertension and 38.2% had diabetes. CREB signaling is important for maintaining homeostasis of glucose metabolism, and activation of CREB signaling is observed in diabetic patients (43). Further, cardiac hypertrophy is strongly associated with hypertension (44). As both signals were suppressed in this study, patient history might have had little influence on the results. Third, 73.5% of the patients in this study had severe COVID-19, and not all cases of the variants were measured. The present results are also based on measurements from a single center and have not been validated in other cohorts. Fourth, many of the supporting rationales for IPA analysis are related to cancer, which tends to lead to cancer-related results. Finally, as blood cell mRNAs are affected by long noncoding RNAs and circular RNAs in addition to blood cell miRNAs, more accurate and detailed evaluation would require mRNA analysis that includes more than just that of blood cell RNAs.

## Conclusion

Integrated analysis of mRNA and miRNAs revealed decreased antigen presentation and T cell refractoriness to antigen presentation in patients with ARDS, resulting in a dysfunctional T cell state. We presented potential mechanisms of how multiple miRNAs are involved in the pathophysiology of ARDS.

## Data availability statement

The data presented in the study are deposited in the NCBI GEO repository, accession number GSE192707.

## Ethics statement

This study involving humans was approved by the institutional review board of Osaka University Hospital (approval number: 885 [Osaka University Critical Care Consortium Novel Omix Project; Occonomix Project]). The studies were conducted in accordance with the local legislation and institutional requirements. The participants provided their written informed consent to participate in this study.

## Author contributions

YM: Conceptualization, Data curation, Formal analysis, Writing – original draft, Writing – review & editing. HM: Conceptualization, Supervision, Writing – review & editing. YT: Data curation, Formal analysis, Writing – review & editing. SaO: Data curation, Formal analysis, Writing – review & editing. ShO: Resources, Writing – review & editing. JY: Resources, Writing – review & editing. AM: Resources, Writing – review & editing. HI: Writing – review & editing. HO: Writing – review & editing. DO: Writing – review & editing. JO: Writing – review & editing.

## Supplementary material

Supplementary Figure 1 Sample size analysis.

Supplementary Figure 2 Results of enrichment analysis based on biological processes and Kyoto Encyclopedia of Genes and Genomes (KEGG) data. **(A)** Dot plot of the top 20 most enriched Gene Ontology (GO) terms for biological processes significantly up- and down-regulated in mRNA. **(B)** Dot plot of the top 20 most enriched among GO terms of biological processes significantly up- and down-regulated in miRNA-targeted mRNAs. **(C)** Dot plot of the top 20 most enriched among KEGG GO terms significantly up- and down-regulated in mRNA. **(D)** Dot plot of the top 20 most enriched GO terms of KEGG that were significantly up- and down-regulated in miRNA-targeted mRNAs. *Reg.* regulation, *proc.* process, *Neg.* Negative.
