# Supplementary figures and images for: T cell dysfunction in elderly ARDS patients based on miRNA and mRNA integration analysis
Source: Front Immunol. 2024 Mar 20;15:1368446. doi: 10.3389/fimmu.2024.1368446 (PMC10987699; doi:10.3389/fimmu.2024.1368446)

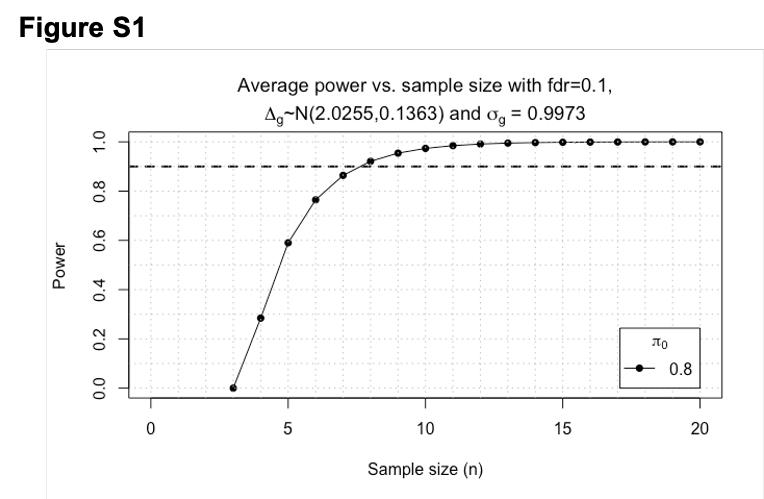

Supplement: Supplementary Figure 1 — Sample size analysis. [file Image_1.tiff]
