# Supplementary material for: T cell dysfunction in elderly ARDS patients based on miRNA and mRNA integration analysis
Source: Front Immunol. 2024 Mar 20;15:1368446. doi: 10.3389/fimmu.2024.1368446 (PMC10987699; doi:10.3389/fimmu.2024.1368446)
Supplement: Supplementary file 2 [file Table_1.docx]

Table S1 RIN value (

Sample No
A_1
A_2
A_4
A_6
A_7
A_8
A_9
A_10
A_13
A_16
A_27
A_33
A_38
A_41
A_43
A_45
A_53
A_57
A_59
A_65
A_68
A_70
A_72
A_73
A_75
A_81
A_86
A_87
A_90
A_97
A_102
A_108
A_112
A_28
H_1
H_2
H_7
H_8
H_9
H_11
H_14
H_15
H_19
H_20
H_21
H_22
H_24
H_25
H_16

RIN; RNA integrity n[illegible]
A; Acute Respiratory
H: Healthy donor
