# Supplementary material for: T cell dysfunction in elderly ARDS patients based on miRNA and mRNA integration analysis
Source: Front Immunol. 2024 Mar 20;15:1368446. doi: 10.3389/fimmu.2024.1368446 (PMC10987699; doi:10.3389/fimmu.2024.1368446)
Supplement: Supplementary file 3 [file Table_2.docx]

Table S2 Significant canonical signaling pathways in mRNA identified in the RNA-seq

Ingenuity Canonical Pathways	-Log(P-value)	z-score
Activated pathways		
PD-1, PD-L1 cancer immunotherapy pathway	6.98	2.887
Interferon Signaling	6.05	3
Role of Hypercytokinemia/hyperchemokinemia in the Pathogenesis of Influenz	3.98	3.545
Inflammasome pathway	3.68	3
MSP-RON Signaling In Macrophages Pathway	3.65	2.646
Neutrophil Extracellular Trap Signaling Pathway	3.43	2.39
Kinetochore Metaphase Signaling Pathway	3.03	2.4
TREM1 Signaling	2.93	2.524
Estrogen-mediated S-phase Entry	2.7	2.121
Toll-like Receptor Signaling	2.46	2.84
Pyroptosis Signaling Pathway	2.29	3.13
CDX Gastrointestinal Cancer Signaling Pathway	2.16	2.333
Role Of Chondrocytes In Rheumatoid Arthritis Signaling Pathway	2.16	2.887
p38 MAPK Signaling	1.93	2.683
Acute Phase Response Signaling	1.8	2.449
Immunogenic Cell Death Signaling Pathway	1.5	2.183
Cyclins and Cell Cycle Regulation	1.43	2.673
iNOS Signaling	1.36	2.53
Inhibited pathways		
Th1 Pathway	12.9	-3.429
Th2 Pathway	11.5	-3.355
S100 Family Signaling Pathway	10.5	-2.506
CREB Signaling in Neurons	6.92	-4.502
Phagosome Formation	6.66	-2.316
Cardiac Hypertrophy Signaling (Enhanced)	6.6	-2.525
Netrin Signaling	5.93	-2.4
G-Protein Coupled Receptor Signaling	5.05	-2.373
GPCR-Mediated Nutrient Sensing in Enteroendocrine Cells	4.97	-2.414
Sperm Motility	4.85	-3
Pulmonary Fibrosis Idiopathic Signaling Pathway	4.35	-2.54
Breast Cancer Regulation by Stathmin1	3.71	-2.222
Oxytocin In Brain Signaling Pathway	3.35	-2.53
Endocannabinoid Neuronal Synapse Pathway	3.31	-2.117
Synaptic Long Term Depression	3.1	-3.212
Role of NFAT in Cardiac Hypertrophy	2.85	-2.271
IL-15 Production	2.7	-3.138
Factors Promoting Cardiogenesis in Vertebrates	2.49	-3.286
Human Embryonic Stem Cell Pluripotency	2.44	-3.124
IL-23 Signaling Pathway	2.25	-2.309
Chronic Myeloid Leukemia Signaling	2.19	-2.188
eNOS Signaling	2.1	-2.357
Basal Cell Carcinoma Signaling	2.08	-2.111
Ferroptosis Signaling Pathway	1.98	-2.041
Thrombin Signaling	1.67	-2.502
Sphingosine-1-phosphate Signaling	1.65	-2.524
Androgen Signaling	1.64	-2.183
Glioblastoma Multiforme Signaling	1.37	-2.041
Neuropathic Pain Signaling In Dorsal Horn Neurons	1.34	-2.357
