# Supplementary material for: T cell dysfunction in elderly ARDS patients based on miRNA and mRNA integration analysis
Source: Front Immunol. 2024 Mar 20;15:1368446. doi: 10.3389/fimmu.2024.1368446 (PMC10987699; doi:10.3389/fimmu.2024.1368446)
Supplement: Supplementary file 4 [file Table_3.docx]

Table S3 Significant canonical signaling pathways in miRNA-targeted mRNA identified in the RNA-seq

Ingenuity Canonical Pathways	-Log(P-value)	z-score
Activated pathways		
PD-1, PD-L1 cancer immunotherapy pathway	2.24	2.828
Inhibited pathways		
Th2 Pathway	7.8	-2.711
CREB Signaling in Neurons	3.13	-2.343
Sperm Motility	2.49	-2.449
cAMP-mediated signaling	1.92	-2.138
IL-15 Production	1.44	-2.53
Cardiac Hypertrophy Signaling	1.42	-3.024
Pulmonary Fibrosis Idiopathic Signaling Pathway	1.42	-2.236
